# Maternal hemoglobin concentrations across pregnancy and child health and development from birth through 6–7 years

**DOI:** 10.3389/fnut.2023.1114101

**Published:** 2023-02-16

**Authors:** Melissa F. Young, Phuong Nguyen, Lan Mai Tran, Long Quynh Khuong, Sonia Tandon, Reynaldo Martorell, Usha Ramakrishnan

**Affiliations:** ^1^Hubert Department of Global Health, Emory University, Atlanta, GA, United States; ^2^Doctoral Program in Nutrition and Health Sciences, Laney Graduate School, Emory University, Atlanta, GA, United States; ^3^Poverty, Health and Nutrition Division, International Food Policy Research Institute (IFPRI), Washington, DC, United States; ^4^Hanoi School of Public Health, Hanoi, Vietnam

**Keywords:** hemoglobin, pregnant women, child development, anemia, nutrition

## Abstract

**Background:**

The role of changes in maternal hemoglobin (Hb) across pregnancy on child health and development (CHD) remains unclear.

**Objective:**

We examined the association between maternal Hb trajectories and CHD outcomes: (a) birth outcomes (birth weight, length, gestational age, preterm, and small for gestational age); (b) child Hb at 3, 6, 12, and 24 months; and (c) motor and mental development at 12 and 24 months and cognitive functioning at age 6–7 years.

**Methods:**

We used data from a randomized controlled trial (PRECONCEPT) conducted in Vietnam (*N* = 1,175 women enrolled during preconception with offspring follow-up through 6–7 years). Maternal Hb trajectories were developed using latent class analysis with Hb data at preconception, early (≤20 weeks), mid (21–29 weeks), and late (≥30 weeks) pregnancy. Multivariable linear and logistic regression models were used to assess the association between maternal Hb trajectories on CHD outcomes, adjusting for confounding variables at the maternal, child and household levels.

**Results:**

Four distinct maternal Hb trajectories were identified. Track 1 (low initial Hb-decline) was associated with lower child Hb at 3 months (β [95% CI] −0.52 [−0.87, −0.16]), 6 months (−0.36 [−0.68, −0.05]), 12 months (−0.46 [−0.79, −0.13]), and 24 months (−0.44 [−0.72, −0.15]) and motor development at 12 months (−3.58 [−6.76, −0.40]) compared to track 4 (high initial Hb-decline). After adjustment for multiple testing, relationships remained robust with the exception of associations with child Hb at 6 months and motor development at 12 months. Track 2 (low initial Hb-improve) was the only Hb trajectory to increase across pregnancy; however, it was insufficiently powered. Track 3 (mid Hb-decline) was associated with lower child Hb at 12 months (−0.27 [−0.44, −0.10]) and 24 months (−0.20 [−0.34, −0.05]) compared to track 4 (high initial Hb-decline). Maternal Hb trajectories were not associated with birth outcomes or child development at 24 months or 6–7 years.

**Conclusion:**

Maternal Hb trajectories during pregnancy are associated with child Hb concentrations across the first 1,000 days, but not with birth outcomes or later cognitive functioning. More work is needed to better understand and interpret changes in Hb levels during pregnancy especially in resource poor settings.

## Introduction

Maternal anemia during pregnancy is a pressing global health problem impacting 32 million women (1). Anemia during pregnancy is associated with increased risk of maternal and neonatal mortality, preterm births (PTB) and small for gestational age (SGA), and impaired child health and development (2–5). The prevention and control of anemia is an important public health priority for meeting the Sustainable Development Goals on nutrition, health and wellbeing, and the WHO 2025 global targets of reducing maternal anemia by 50% (4, 6).

Despite the widespread acknowledgment of the importance of preventing anemia during pregnancy to improve maternal and child health outcomes (7, 8), many questions remain. A systematic review by Young et al. (5), identified several limitations of existing literature in response to a special call by the WHO to review global guidelines for anemia. First, although prior research suggests a differential impact of maternal hemoglobin (Hb) concentrations during pregnancy on birth outcomes depending on timing of measurement (i.e., gestational age), there are few longitudinal studies with serial measurements of maternal Hb over the course of pregnancy and/or reliable estimates of gestational age (5, 9). Defining anemia during pregnancy is further complicated by the dramatic increases in plasma volume expansion (10), Early in pregnancy, maternal hemoglobin may play a key role in placental development and function as well as nutrient availability for fetal growth and development, whereas mid pregnancy is a critical time period of rapid fetal growth and late pregnancy is a time period of greatest placental iron transfer to fetus and completion of organ development (11–14). Second, most studies do not use valid methods to evaluate the independent and time-specific relative contributions of Hb levels, which tend to be highly correlated across pregnancy and early childhood. Third, few prospective cohorts examine the relationship between maternal Hb and long-term child development outcomes. Despite decades of research, the relationship between maternal Hb concentrations and long-term child development remains unclear (5, 15). Furthermore, there is limited data that has examined these associations in contexts with a high prevalence of anemia but low prevalence of iron deficiency anemia.

In order to address these important research gaps, we leveraged secondary data from a large micronutrient supplementation trial conducted in Vietnam (PRECONCEPT, NCT01665378) (16). Over 5,000 non-pregnant women who were intending to get pregnant were enrolled and those who conceived were carefully monitored throughout pregnancy and their offspring were followed from birth through age 6–7 years. Our objective was to examine the association between maternal Hb trajectories during pregnancy and child health and development outcomes: (a) birth outcomes (birth weight, length, gestational age, preterm, and small for gestational age); (b) child Hb at 3 months, 6 months, 12 months, and 24 months; and (c) motor and mental development at 12 months and 24 months and cognitive functioning at age 6–7 years.

## Materials and methods

### Study design, participants, and setting

Children in this study are offspring of women who participated in a randomized controlled trial that was originally designed to evaluate the effects of preconception micronutrient supplementation on maternal and child health outcomes in Vietnam (PRECONCEPT study; NCT: 01665378). Details of the PRECONCEPT study have been published previously (16). Briefly, the study included 5,011 women of reproductive age who were randomly assigned to receive weekly supplements containing either 2,800 μg folic acid (FA), 60 mg iron and 2,800 μg FA (IFA), or multiple micronutrients (MM) containing the same amount of IFA, from baseline until conception, followed by daily prenatal supplements containing 60 mg iron and 400 μg FA until delivery. Women were followed prospectively to identify pregnancies and evaluate child outcomes from delivery through 6–7 years. We included women with singleton, live births and data on birth weight, gestational age as well as maternal Hb data during preconception and three time points during pregnancy.

### Outcome measures

The key outcomes of interest include: (1) birth outcomes; (2) offspring Hb during the first 1,000 days; and (3) child development at 12 months, 24 months and 6–7 years described in brief below and in further depth in prior publications (16–19).

#### Birth outcomes

Birth weight was measured as early as possible within 7 days after birth using standard procedures and highly trained research assistants (20) using electronic weighing scales precise to 10 g. Birth length was measured with collapsible length boards, which were precise to 1 mm. Gestational age was calculated as the number of days between the first day of the last menstrual period (obtained prospectively by village health workers during their biweekly home visits) and the day of delivery. Prior work has reported on accuracy and validity of estimates compared to ultrasound measurements in this study (21). A pre-term birth was defined as a birth occurring before 37 completed weeks of pregnancy. Small for gestational age (SGA) was defined as a birth weight below the 10th percentile for gestational age and sex (22).

#### Offspring Hb during the first 1,000 days

Offspring Hb was measured from finger prick capillary blood samples at 3 months, 6 months, 12 months, and 24 months of age using a portable HemoCue 301 Analyzer (23). Child anemia was defined as a Hb value < 11 g/dL (24). All research assistants were highly trained with close supervision on standard protocols (16, 19).

#### Child development

Child development at 12 and 24 months of age was assessed using the Bayley Scales of Infant Development (BSID) III (25) which includes cognitive, language, and motor subscales. The BSID-III has been translated and adapted in Vietnam using standardized methods and has been used in previous studies (26–28). The raw summary scores for each of the domains were then transformed to standardized composite scores (approximately mean ± SD: 100 ± 15) to facilitate comparisons across domains. The BSID-III was administered in a quiet room at community health centers by well-trained researchers. Data quality was assessed based on weekly field-based supervision and monthly staff meetings. Site visits were also carried out regularly by study investigators, and refresher training sessions were conducted every 6 months after the initial training to ensure testing was conducted in a standardized manner.

Child intellectual development at 6–7 years was assessed using the Wechsler Intelligence Scale for Children^®^—Fourth Edition (WISC–IV) (29). The WISC-IV consists of four specific cognitive domains (Verbal Comprehension Index–VCI, Perceptual Reasoning Index–PRI, Working Memory Index–WMI, and Processing Speed Index–PSI) and the Full-Scale Intelligence Quotient (FSIQ). The WISC–IV has been translated, adapted, and validated to be a standardized test in Vietnam (30). The WISC-IV was assessed by pediatricians or researchers with master’s degrees in public health. Quality control were conducted with field-based supervision, monthly staff meetings, and refresher training (18).

### Exposure variables

The primary exposure variables are Hb at preconception, early (≤20 weeks), mid (21–29 weeks), and late (≥30 weeks) pregnancy that were measured by Hemocue™ 301 from a capillary blood sample obtained by finger-prick (23) using standardized training protocols (19).

### Confounders

Confounding variables were considered at child, maternal, and household levels. These included child sex, maternal age, maternal education, maternal depression, and intervention group. Maternal education was categorized into four groups: primary school (completed 1–5 years), secondary school (6–9 years), high school (10–12 years), and college or higher. Maternal depression was measured at baseline using the Center for Epidemiologic Studies Depression Scale (CES-D) (31). The intervention group included the FA, IFA, and MM groups. At household level, the quality of the learning environment at home was measured using the Infant/Toddler HOME inventory at 12 months of age (32); the HOME assesses the quality and quantity of the social, emotional, and cognitive support available to a child in the home environment. Household socio-economic status (SES) index was calculated using a principal components analysis of housing quality and assets that were assessed at baseline; the first component derived from component scores was used to divide household SES into tertiles (33, 34).

### Statistical analysis

The Kolmogorov–Smirnov test was used for testing the normality of the continuous outcome variables. Descriptive statistics were used to report characteristics of the study population, with frequencies and percentages to describe categorical variables, means, and standard deviations to describe quantitative variables.

We used Latent Class Growth Analysis (LCGA) to identify different maternal Hb latent classes with qualitatively distinct trajectories. Trajectories of maternal Hb was created using censored normal model which was most appropriate for the ordinal nature of the indicator variables, Hb level at preconception early (≤20 weeks), mid (21–29 weeks), and late (≥30 weeks) pregnancy in order to allow for us to maximize sample size and equally distribute available data over critical periods across pregnancy. We selected best fitting model in identifying the tracks by fitting different models with the increasing numbers of trajectories. The final number of trajectories were determined based on the information criteria (AIC: Akaike’s Information Criterion; BIC: Bayesian Information Criterion) and the interpretability of the class membership. The LCGA categorized women into four Hb trajectories, in which no additional variation in the estimated trajectory within class, just random error.

Multivariable linear regressions (for continuous outcomes) and logistic regression (for binary outcomes) were used to assess the association between maternal Hb trajectories and birth outcomes, offspring Hb during the first 1,000 days, and child development at 12 months, 24 months and 6–7 years. Associations with birth outcomes and offspring Hb during the first 1,000 days were examined in two models: (1) unadjusted model; and (2) adjusted for maternal age, ethnicity, education, SES, infant sex, and age and intervention group. Similar analyses were conducted to examine associations with child development at 12 months and 24 months and child intellectual development at 6–7 years with additional adjustment for home environment, mother IQ, and depression. All analyses were conducted using Stata v17 (StataCorp, College Station, TX, USA). An initial significance level of 0.05 was used for all statistical tests and adjusted for multiple testing using the Bonferroni Approach for each domain outcome.

### Ethical approval

The study was approved by the Ethical Committee of Institute of Social and Medicine Studies in Vietnam and Emory University’s Institutional Review Board, Atlanta, Georgia, USA. The trial was registered in the US Clinical Trials registry (identification number NCT01665378). Written informed consent was obtained from all study participants.

## Results

**FIGURE 1:**
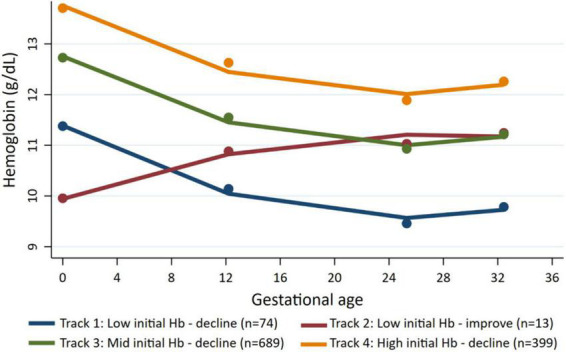
Maternal Hb trajectories across pregnancy.

We identified four distinct maternal Hb trajectories (Figure 1 and Supplementary Table 1). Track 1 (named *low initial Hb-decline*) included women with low initial mean Hb (11.1 ± 1.0 g/dL) at preconception and declined (mean Hb between 9.3–9.6 g/dL) during pregnancy (*n* = 74, 7.5%). Track 2 (named *low initial Hb-improve*) included women with the lowest initial mean Hb (9.5 ± 1.2 g/dL) at preconception; however, Hb recovered early in pregnancy and remained between 10.6–11.5 g/dL throughout pregnancy (*n* = 13, 1.7%). Track 3 (named *mid initial Hb-decline*) included women with an initial mean Hb of 12.7 ± 1.0 and remained between 10.9–11.5 g/dL (*n* = 689, 55%). Track 4 (named *high initial Hb-decline*) include women with high preconception Hb (13.9 ± 1.1 g/dL) and remained between 12.0–12.8 g/dL (*n* = 399, 36%).

Preconception baseline characteristics of the women by the different Hb trajectories are described in Table 1. Maternal age and education were similar across the four groups with an average age around 26 year and over half of the women completed secondary school. Maternal BMI was likewise similar across groups with 30% of women having low BMI; the exception was Track 2 (*low initial Hb-improve*) where almost 70% of women had a low BMI. Track 1 (*low initial Hb-decline*) and Track 3 (*mid initial Hb-decline*) had a greater prevalence of minority women and employment in farming. Child characteristics are described in Table 2. Overall, 8.5% of infants were born preterm, 4.4% were born low birth weight and 15.5% were born SGA. Child anemia was high with over 60% anemia at 3 months, 53% at 12 months, and 40% at 24 months. Basic maternal and child characteristics were also similar for the final analytic sample of 1,175 mother-child pairs (Supplementary Figure 1) when compared to those who were excluded due to missing data (Supplementary Tables 2, 3).

**TABLE 1:** Baseline characteristics of participants at preconception enrollment, by maternal Hb trajectories^1^.

Variable	All women (*n* = 1,175)	Track 1 (*n* = 74)	Track 2 (*n* = 13)	Track 3 (*n* = 689)	Track 4 (*n* = 399)
Age (years)	26.0 ± 4.3	26.5 ± 4.8	27.8 ± 3.4	25.8 ± 4.4	26.1 ± 4.1
Minority ethnic, %^2^	577 (49.2)	46 (62.2)	6 (46.2)	359 (52.2)	166 (41.7)
Work as farmers, %^2^	938 (79.9)	61 (82.4)	10 (76.9)	575 (83.5)	292 (73.4)
Number of children ≥1, %	927 (94.2)	62 (96.9)	10 (100.0)	546 (94.6)	309 (92.8)
Time to pregnancy	33.8 ± 24.3	33.6 ± 25.9	29.0 ± 28.9	34.3 ± 24.4	33.1 ± 23.7
**Education level, %**					
Primary school	88 (7.5)	4 (5.4)	2 (15.4)	52 (7.5)	30 (7.5)
Secondary school	636 (54.2)	39 (52.7)	7 (53.8)	396 (57.5)	194 (48.7)
High school	305 (26.0)	23 (31.1)	3 (23.1)	169 (24.5)	110 (27.6)
College or higher	145 (12.4)	8 (10.8)	1 (7.7)	72 (10.4)	64 (16.1)
**Socio-economic status, %**					
Low	385 (32.8)	31 (41.9)	3 (23.1)	228 (33.1)	123 (30.9)
Average	393 (33.5)	24 (32.4)	4 (30.8)	239 (34.7)	126 (31.7)
High	396 (33.7)	19 (25.7)	6 (46.2)	222 (32.2)	149 (37.4)
**Preconception nutritional status**					
Weight (kg)	45.7 ± 5.3	45.3 ± 4.9	44.1 ± 5.2	45.6 ± 5.1	46.1 ± 5.8
Height (cm)	152.6 ± 5.0	152.9 ± 5.0	153.7 ± 5.7	152.3 ± 5.0	153.0 ± 5.0
BMI (kg/m^2^)	19.6 ± 2.0	19.4 ± 1.9	18.7 ± 2.2	19.6 ± 1.8	19.7 ± 2.2
Low BMI (<18.5), %	359 (30.6)	22 (29.7)	9 (69.2)	201 (29.2)	127 (32.0)
Preconception Hb (g/dl^2^)	12.9 ± 1.3	11.1 ± 1.0	9.5 ± 1.2	12.7 ± 1.0	13.9 ± 1.1
Anemia, % (Hb < 12 g/dL)^2^	236 (20.1)	58 (78.4)	12 (92.3)	151 (21.9)	15 (3.8)
Mild (11 < Hb <12 g/dL)	164 (14.0)	29 (39.2)	0 (0.0)	121 (17.6)	14 (3.5)
Moderate/severe (Hb <11 g/dL)	72 (6.1)	29 (39.2)	12 (92.3)	30 (4.4)	1 (0.3)

^1^Values are means ± SDs or *n* (%). BMI, body mass index; FA, folic acid; Hb, Hb; IFA, iron and folic acid; MM, multiple micronutrient; SGA, small for gestational age.

^2^Significant differences across the four Hb groups, *p* < 0.05.

**TABLE 2:** Child characteristics at birth, Hb through 1st 1,000 days and child development through 6–7 years^1^.

Variable	*n* = 1,175
**Child characteristics**	
Female, %	584 (49.7)
Gestational age (week)	39.3 ± 1.9
Preterm, %	100 (8.5)
Birth weight (gr)	3,094.4 ± 439.7
Low birth weight, %	52 (4.4)
SGA, %	172 (15.5)
Birth length (cm)	48.9 ± 3.1
Child Hb	
Hb at 3 months (g/dl)	10.6 ± 1.3
Anemia, % (Hb <11 g/dL)	615 (61.0)
Hb at 12 months (g/dl)	10.8 ± 1.2
Anemia, % (Hb <11 g/dL)	486 (52.7)
Hb at 24 months (g/dl)	11.2 ± 1.2
Anemia,% (Hb <11 g/dL)	209 (39.9)
**Bayley scales for infant development at 12 months**	
Cognitive	112.2 ± 10.3
Language	97.8 ± 11.0
Motor	102.9 ± 11.7
Bayley scales (overall)	104.3 ± 8.7
**Bayley scales for infant development at 24 months**	
Cognitive	99.3 ± 9.8
Language	102.2 ± 11.0
Motor	105.8 ± 12.1
Bayley scales (overall)	102.9 ± 8.9
**Wechsler scale global intelligence, performance and verbal scores at 6–7 years**	
Verbal comprehension index (VCI)	82.0 ± 12.4
Perceptual reasoning index (PRI)	93.5 ± 14.2
Working memory index (WMI)	101.8 ± 11.6
Processing speed index (PSI)	89.6 ± 12.4
Full scale IQ (FSIQ)	88.6 ± 12.2

^1^Values are means (SDs) or *n* (%).

BMI, body mass index; FA, folic acid; Hb, Hb; IFA, iron and folic acid; MM, multiple micronutrient; SGA, small for gestational age.

Maternal Hb trajectories were not significantly associated with birth weight, birth length, gestational age, preterm and SGA (Table 3). Maternal Hb trajectories were significantly associated with offspring Hb during the first 1,000 days (Table 4). Offspring of women in Track 1 (*low initial Hb-decline*) had lower Hb at ages 3 months (β [95% CI] −0.52 [−0.87, −0.16]), 6 months (−0.36 [−0.68, −0.05]), 12 months (−0.46 [−0.79, −0.13]), and 24 months (−0.44 [−0.72, −0.15]) compared to those born to women in track 4 (*high initial Hb-decline*). Children born to women in Track 3 (*mid initial Hb-decline*) also had lower Hb at 12 months (−0.27 [−0.44, −0.10]) and 24 months (−0.20 [−0.34, −0.05]) compared to those born to women in track 4. These results remained significant after applying a more stringent *p*-value of 0.01 to account for multiple testing with the exception for associations with child Hb at 6 months which became non-significant.

**TABLE 3:** Maternal Hb trajectories and association with birth outcomes^1^.

Variable	Birth weight (β)		Birth length (β)		GA (β)		Preterm (OR)		SGA (OR)	
	Unadjusted	Adjusted	Unadjusted	Adjusted	Unadjusted	Adjusted	Unadjusted	Adjusted	Unadjusted	Adjusted
Track 1 (low initial Hb-decline)	−94.10^+^ [−203.22, 15.02]	−86.17 [−195.42, 23.08]	0.44 [−0.38, 1.26]	0.42 [−0.40, 1.25]	0.26 [−0.20, 0.72]	0.16 [−0.30, 0.62]	0.89 [0.36, 2.19]	1.03 [0.41, 2.58]	1.57 [0.84, 2.93]	1.62 [0.86, 3.06]
Track 2 (low initial Hb-improve)	−107.60 [−350.59, 135.39]	−127.01 [−369.20, 115.18]	−1.15 [−2.96, 0.66]	−1.25 [−3.06, 0.57]	−0.11 [−1.13, 0.92]	−0.12 [−1.14, 0.90]	0.84 [0.11, 6.65]	0.79 [0.10, 6.45]	1.68 [0.45, 6.28]	2.04 [0.53, 7.81]
Track 3 (mid initial Hb-decline)	9.83 [−44.41, 64.07]	15.56 [−39.03, 70.14]	0.30 [−0.11, 0.71]	0.31 [−0.10, 0.73]	0.04 [−0.19, 0.27]	−0.03 [−0.26, 0.20]	0.91 [0.59, 1.41]	0.98 [0.62, 1.53]	0.98 [0.68, 1.39]	1.00 [0.69, 1.43]
*N*	1,175	1,172	1,072	1,070	1,175	1,172	1,175	1,172	1,108	1,105

^1^Track 4 (*high initial Hb-decline*) is the reference category; Values are (β [95% CI]) or (OR [95% CI]).

Models adjusted for maternal ethnicity, age, education, SES, infant sex, intervention group. GA, gestational age, SGA, small for gestational age.

**TABLE 4:** Association between maternal Hb trajectories and offspring Hb during the first 1,000 days^1^.

Variable	Hb 3 months		Hb 6 months		Hb 12 months		Hb 24 months	
	Unadjusted	Adjusted	Unadjusted	Adjusted	Unadjusted	Adjusted	Unadjusted	Adjusted
Track 1 (low initial Hb-decline)	−0.49 [−0.85, −0.14]	−0.52 [−0.87, −0.16]	−0.35 [−0.67, −0.04]	−0.36 [−0.68, −0.05]	−0.51 [−0.84, −0.18]	−0.46 [−0.79, −0.13]	−0.48 [−0.76, −0.19]	−0.44 [−0.72, −0.15]
Track 2 (low initial Hb-improve)	−0.25 [−1.00, 0.51]	−0.32 [−1.08, 0.43]	−0.08 [−0.76, 0.60]	0.01 [−0.66, 0.69]	−0.37 [−1.10, 0.35]	−0.37 [−1.08, 0.34]	0.23 [−0.37, 0.84]	0.22 [−0.38, 0.83]
Track 3 (mid initial Hb-decline)	−0.10 [−0.27, 0.08]	−0.10 [−0.28, 0.07]	−0.14 [−0.30, 0.01]	−0.12 [−0.28, 0.03]	−0.31 [−0.48, −0.15]	−0.27 [−0.44, −0.10]	−0.22 [−0.37, −0.08]	−0.20 [−0.34, −0.05]
*N*	1,007	1,005	960	960	943	943	1,031	1,030

^1^Track 4 (*high initial Hb-decline*) is the reference category; Values are (β [95% CI]).

Models adjusted for maternal ethnicity, age, education, SES, infant age and sex, intervention group. Hb, hemoglobin.

Offspring born to women in Track 1 (*low initial Hb-decline*) had lower motor development scores at 12 months (−3.58 [−6.76, −0.40]) when compared to track 4 (*high initial Hb-decline*) (Table 5). However, this association was no longer significant after adjusting for multiple comparisons (0.05 > *p* > 0.017) and was not observed at 24 months. Maternal Hb trajectories were also not associated with child cognition or language at 12 or 24 months or with child cognition at 6–7 years in adjusted models (Table 6).

**TABLE 5:** Association between maternal Hb trajectories and child development at 12 and 24 months^1^.

	Child development–12 months					
	Cognition–12 months		Language–12 months		Motor 12 months	
Variable	Unadjusted	Adjusted	Unadjusted	Adjusted	Unadjusted	Adjusted
Track 1 (low initial Hb-decline)	−1.63 [−4.45, 1.18]	−1.25 [−4.09, 1.58]	−0.54 [−3.54, 2.47]	−0.67 [−3.68, 2.35]	−3.75 [−6.95, −0.55]	−3.58 [−6.76, −0.40]
Track 2 (low initial Hb-improve)	2.30 [−3.65, 8.25]	2.25 [−3.66, 8.16]	−0.82 [−7.17, 5.53]	−1.14 [−7.44, 5.15]	−0.60 [−7.35, 6.16]	−0.66 [−7.29, 5.98]
Track 3 (mid initial Hb-decline)	−0.69 [−2.06, 0.69]	−0.29 [−1.69, 1.12]	−0.89 [−2.35, 0.58]	−0.77 [−2.27, 0.73]	−0.99 [−2.56, 0.57]	−0.72 [−2.30, 0.86]
*N*	1,008	969	1,009	970	1,008	969
	**Child development–24 months**					
	**Cognition–24 months**		**Language–24 months**		**Motor 24 months**	
**Variable**	**Unadjusted**	**Adjusted**	**Unadjusted**	**Adjusted**	**Unadjusted**	**Adjusted**
Track 1 (low initial Hb-decline)	−0.56 [−3.05, 1.93]	−0.17 [−2.63, 2.30]	0.13 [−2.69, 2.94]	0.40 [−2.37, 3.17]	1.58 [−1.52, 4.68]	1.93 [−1.16, 5.02]
Track 2 (low initial Hb-improve)	4.87^+^ [−0.54, 10.28]	4.73^+^ [−0.60, 10.05]	0.76 [−5.32, 6.84]	0.79 [−5.16, 6.75]	0.31 [−6.39, 7.01]	−0.47 [−7.12, 6.18]
Track 3 (mid initial Hb-decline)	−0.08 [−1.34, 1.18]	0.32 [−0.93, 1.58]	−0.64 [−2.06, 0.78]	−0.20 [−1.60, 1.20]	−0.79 [−2.35, 0.77]	−0.54 [−2.10, 1.03]
*N*	1,084	1,080	1,082	1,077	1,081	1,076

^1^Track 4 (high initial Hb-decline) is the reference category; Values are (β [95% CI]).

Models adjusted for maternal ethnicity, age, education, SES, infant age and sex, intervention group, home environment, maternal IQ, depression.

**TABLE 6:** Association between maternal Hb trajectories and child cognition at 6–7 years^1^.

Variable	VCI		PRI		WMI		PSI		FSIQ	
	Unadjusted	Adjusted	Unadjusted	Adjusted	Unadjusted	Adjusted	Unadjusted	Adjusted	Unadjusted	Adjusted
Track 1 (low initial Hb-decline)	−1.70 [−5.12, 1.72]	−0.85 [−4.27, 2.57]	−2.01 [−5.93, 1.91]	−0.20 [−4.08, 3.68]	−0.76 [−3.96, 2.44]	−0.64 [−3.86, 2.58]	−1.79 [−5.20, 1.62]	−1.32 [−4.73, 2.09]	−2.13 [−5.49, 1.23]	−0.94 [−4.24, 2.35]
Track 2 (low initial Hb-improve)	1.21 [−6.25, 8.68]	0.75 [−6.59, 8.10]	−6.23 [−14.79, 2.34]	−6.50 [−14.83, 1.82]	−1.80 [−8.80, 5.19]	−2.86 [−9.77, 4.06]	2.07 [−5.37, 9.52]	1.48 [−5.83, 8.80]	−1.81 [−9.15, 5.53]	−2.47 [−9.55, 4.61]
Track 3 (mid initial Hb-decline)	−2.06 [−3.74, −0.37]	−1.09 [−2.79, 0.61]	−0.89 [−2.83, 1.04]	0.42 [−1.51, 2.35]	−0.42 [−2.01, 1.16]	−0.06 [−1.67, 1.55]	−1.33 [−3.02, 0.35]	−0.58 [−2.28, 1.12]	−1.57 [−3.23, 0.09]	−0.41 [−2.06, 1.23]
*N*	969	947	969	947	967	945	969	947	967	945

^1^Track 4 (high initial Hb-decline) is the reference category; Values are (β [95% CI]).

Models adjusted for maternal ethnicity age, education, SES, infant age and sex, intervention group, home environment, and mother depression. VCI, verbal comprehension index; PRI, perceptual reasoning index; WMI, working memory index; PSI, processing speed index; FSIQ, the full-scale intelligence quotient.

## Discussion

In this cohort from Vietnam, we identified four unique maternal Hb trajectories across pregnancy which were not a significant predictor of birth outcomes, but were associated with child Hb across the first 1,000 days of life. Maternal Hb trajectories were not associated with long-term child development or cognition at age 6–7 years.

The lack of association between maternal Hb trajectories during pregnancy and birth outcomes are in contrast to prior research demonstrating the importance of maternal Hb concentrations on adverse birth outcomes (5, 9). There are several factors that may contribute to our findings. First, 80% of women entering pregnancy were non-anemic and most of those who were anemic had mild anemia. However, there was divergence in Hb concentrations patterns across pregnancy and although we had hypothesized that track 1 (*low initial Hb-decline*) would have the highest risk of adverse birth outcomes, this was not the case. This could likewise be due in part to the nature of anemia in our cohort and low prevalence of iron deficiency anemia (19, 35). However, it remains unclear how the etiology of anemia may impact birth outcomes (5). Furthermore, all the women in our study were randomized to receive weekly supplements containing IFA, MM or FA preconception, followed by daily IFA supplementation during pregnancy. Compliance was high with nearly 80% of women consuming more than 80% of the preconception and prenatal tablets (36). While preconception supplementation with IFA and MM was not associated with improvements in birth outcomes or anemia reduction in this cohort, modest improvements in maternal and infant iron status were noted (17, 19). In addition, IFA supplementation during pregnancy is a well-established public health intervention associated with lowered risk of maternal anemia and improved birth outcomes (7, 37). It is possible the high overall uptake of an evidence-based intervention among all participants may have decreased the likelihood of demonstrating associations with maternal Hb on birth outcomes. High maternal Hb during pregnancy likewise has been associated with adverse birth outcomes (5, 9); potentially driven by inadequate plasma volume expansion (38) or excess iron (39–41). Among women in track 4 (*high initial Hb-decline*), nearly 40% had Hb values greater than 13 g/dL in early pregnancy and nearly 25% in late pregnancy. However, this track was not associated with increased risk of adverse birth outcomes. Further research examining the etiology of anemia and high Hb throughout pregnancy is needed to better understand the underlying mechanisms. Furthermore, other nutritional and non-nutritional risk factors should be explored to help guide future programs and improve birth outcomes in this context.

Maternal Hb trajectories were associated with child Hb across the first 1,000 days of life. In particular, for offspring born to women in track 1 (*low initial Hb-decline*) of which over 90% were anemic throughout pregnancy, had lower Hb concentrations at 3, 6, 12, and 24 months compared to those born to women in track 4 (*high initial Hb-decline*). The largest effect sizes were noted at 3 months (β [95% CI] −0.52 [−0.87, −0.16]), but remained high even at 24 months (−0.44 [−0.72, −0.15]), and remained significant even after adjustment for multiple testing with the exception of the 6-months measurement. This could be due to the greater variability in diet and other risk factors during the transition to complementary foods. For women in track 3 (*mid Hb-decline*), associations between maternal and child Hb were significant only at 12 and 24 months. The rationale for the delayed associations is unclear, though this could be associated with the underlying etiology of anemia. While up to 50% of maternal anemia is often attributed to iron deficiency, this can vary widely across different contexts (4, 42). Prior research has suggested a potentially stronger correlation between maternal iron deficiency anemia with child anemia compared to maternal non-iron deficiency anemia (43). Maternal iron deficiency anemia is associated with decreased iron endowment at birth and thus greater risk of early infant anemia (44). However, in our cohort, preconception iron deficiency was very low (<5%) and maternal anemia may be more driven by other factors such as hookworm infections (19, 35). Shared dietary, genetic (hemoglobinopathies) or environmental factors could contribute to delayed as well as continued associations across early childhood. The role of other micronutrient deficiencies, such as vitamins A and B_12_, during pregnancy and their potential influence on infant hemoglobin likewise remains unclear. A better understanding of the etiology of maternal anemia and impact on child hematologic and micronutrient profiles is needed. Furthermore, few studies have examined how the strength of association between maternal and child Hb may vary by timing of assessment. In a recent 2020 systematic review among 32 studies, there was a negligible association between maternal Hb and offspring Hb within the first 48 h of life (0.15: 95% CI 0.10, 0.20) (45). The authors note the heterogenous and weak associations may be due to varying anemia etiology across included studies (45).

We had expected that maternal Hb trajectories would be associated with child development; however, we found only modest associations with motor development at 12 months and no long-term associations at 6–7 years. In the literature, few studies have examined the long-term impact of maternal anemia on functional outcomes such as offspring IQ or school readiness (46, 47). A recent study of a Danish cohort of over 500,000 children showed that anemia in early pregnancy was associated with over a twofold increased risk of development of intellectual disability in the offspring between the ages of 6–29 years, but much weaker associations were seen for anemia in late pregnancy (48). In a prospective multicenter cohort study among 12 university hospitals in the US, maternal hematocrit during pregnancy was associated with offspring IQ at 4 and 7 years of age (46). The lack of long-term impact in our cohort of maternal Hb trajectories on child development could be due to similar reasons as discussed for birth outcomes: low prevalence of iron deficiency anemia early in pregnancy and primarily mild anemia, etiology of anemia, strong adherence to prenatal supplementation, or other unknown factors. Further replication of our findings in contexts with a higher burden of anemia and micronutrient deficiencies may be merited.

A key strength of our study is the use of prospectively collected data with low loss to follow up that allowed us to comprehensively examine changes in maternal and child Hb across the continuum of preconception and pregnancy through early childhood at 6–7 years. This is an advantage over prior research that has often examined associations focusing on women of reproductive age, pregnancy, or childhood in isolation. Our study had over 95% power, at an alpha of 5%, to detect biologically relevant associations between maternal Hb and child health and development indicators. Another important strength is the availability of repeated measurements of maternal Hb before and during pregnancy and accurate assessment of gestational age (16), which is rare yet essential for current analysis given the effects of plasma volume expansion during pregnancy. Another key strength was our trajectory analytic approach that allows us to leverage multiple highly correlated Hb measures across pregnancy and examine associations with CHD.

While our study provides rich prospective data on child health and development, a limitation of this work is insufficient data on maternal health outcomes. Prior work using a similar Hb trajectory approach, reported that women with low Hb across pregnancy or who had a progressively decreasing Hb trajectory were at a higher risk of transfusion (49). On the other hand, in this cohort high maternal Hb trajectories were associated with increased risk of preterm birth and SGA (49). Thus, demonstrating a need for future work to have dual focus on both maternal and child outcomes as well consideration of low and high Hb levels. Another limitation is that while we had a relatively large overall sample size of women, track 2 (*low initial Hb-improve*) was underpowered and no conclusions can be drawn on this trajectory. We ran sensitivity analyses excluding the women in this track and it does not impact our key findings (data not shown). While we cannot draw conclusions on the association of track 2 Hb trajectories and CHD, we opted to retain all data in the manuscript to show the full picture. For all trajectories, other than track 2, preconception Hb values set the stage for pregnancy and then concentrations decreased throughout pregnancy. Track 2 was the only trajectory to increase during the course of pregnancy. Our study also used capillary blood samples rather than venous blood samples for assessing hemoglobin and this may increase measurement error (50). Another limitation of this work is a lack of data on the etiology of anemia during pregnancy. Further research on the complex and context specific nutritional and non-nutritional risk factors is needed to better understand key findings as well plan appropriate public health interventions. Our findings from a south-eastern Asian population on the four Hb trajectories identified may not be generalizable to other contexts with lower IFA compliance or a greater iron deficiency burden. Further research is needed to identify and examine the impact of maternal Hb trajectories in other contexts.

Our study provides novel insight on the importance of maternal Hb during pregnancy for CHD outcomes. While maternal Hb trajectories were associated child Hb concentrations across the first 2 years of life, we did not find associations with birth outcomes or long-term child development. We also found that maternal Hb trajectories across pregnancy were largely set by preconception Hb concentrations, and the continued monitoring of Hb across pregnancy added limited further insight compared to the effects of preconception Hb alone. These findings indicate the need for early targeting of women before pregnancy in this context. Our findings also indicate the need to examine the role of the etiology of anemia to better understand the underlying mechanisms and context specific findings. Future research may be merited to examine interventions above and beyond IFA supplementation during pregnancy to optimize maternal Hb and child health and development outcomes across different contexts.

## Data availability statement

The data analyzed in this study is subject to the following licenses/restrictions: Data available upon request and approval of study team. Requests to access these datasets should be directed to UR, uramakr@emory.edu and PN, p.h.nguyen@cgiar.org.

## Ethics statement

The studies involving human participants were reviewed and approved by the Ethical Committee of the Institute of Social and Medicine Studies in Vietnam and Emory University’s Institutional Review Board, Atlanta, Georgia, USA. Written informed consent to participate in this study was provided by the participants’ legal guardian/next of kin.

## Author contributions

MY, PN, RM, and UR designed the research. PN, LT, and LK conducted the field research. MY, LT, LK, ST, and PN analyzed the data. MY, PN, and UR wrote the manuscript. MY had primary responsibility for the final content of the manuscript. All authors reviewed and provided critical feedback.
