# Supplementary material for: Maternal hemoglobin concentrations across pregnancy and child health and development from birth through 6–7 years
Source: Front Nutr. 2023 Feb 16;10:1114101. doi: 10.3389/fnut.2023.1114101 (PMC9978095; doi:10.3389/fnut.2023.1114101)
Supplement: Supplementary file 1 [file Table_1.docx]

Supplementary Material

Maternal hemoglobin concentrations across pregnancy and child health and development from birth through 6-7 y.

Melissa F. Young^1,2^, Phuong Nguyen^3^, Lan Mai Tran^1,2^, Long Quynh Khuong^4^, Sonia Tandon^1,2^, Martorell, Reynaldo^1,2^, Usha Ramakrishnan^1,2^

^1^Hubert Department of Global Health, Emory University, Atlanta, GA, USA

^2^Doctoral Program in Nutrition and Health Sciences, Laney Graduate School, Emory University, Atlanta, GA

^3^Poverty, Health and Nutrition Division, International Food Policy Research Institute (IFPRI), Washington, DC, USA

^4^Hanoi School of Public Health, Hanoi, Vietnam

*** Correspondence:**Melissa F. Young, PhD

Emory University

Hubert Department of Global Health Department

1518 Clifton Road, Atlanta, GA

404-727-1529

[melissa.young@emory.edu](mailto:melissa.young@emory.edu)

# Supplementary Figures and Tables

**OSM Figure 1: Flow chart for analytical sample**

**
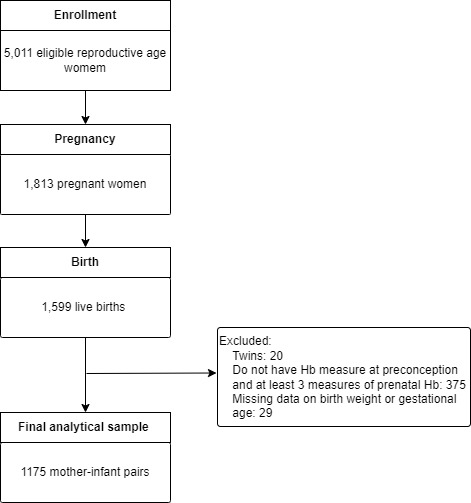
**

**OSM Table 1: Basic descriptive for the 4 tracks at each time point**

| **Variable** | **Track 1**  **(low initial Hb-decline)**  ***(n = 74, 7.5%)*** | **Track 2**  **(low initial Hb-improve)**  **(n=13, 1.7%)** | **Track 3**  **(mid initial Hb-decline)**  **(n=689, 55%)** | **Track 4**  **(high initial Hb-decline)**  **(n=399, 36%)** |
| --- | --- | --- | --- | --- |
| Hb, g/dl |  |  |  |  |
| Preconception | 11.1 (1.0) | 9.5 (1.2) | 12.7 (1.0) | 13.9 (1.1) |
| Early (≤20 wks) pregnancy | 9.9 (0.9) | 10.6 (1.7) | 11.5 (1.0) | 12.8 (1.0) |
| Mid (21-29 wks) pregnancy | 9.3 (1.0) | 11.3 (0.7) | 10.9 (0.9) | 12.0 (0.9) |
| Late (≥30 wks) pregnancy | 9.6 (1.0) | 11.5 (0.9) | 11.2 (1.0) | 12.4 (1.0) |
| Anemia, *%* |  |  |  |  |
| Preconception | 58 (78.4) | 12 (92.3) | 151 (21.9) | 15 (3.8) |
| Early (≤20 wks) pregnancy | 65 (90.3) | 7 (53.8) | 178 (27.1) | 14 (3.7) |
| Mid (21-29 wks) pregnancy | 68 (95.8) | 3 (27.3) | 330 (51.1) | 48 (12.7) |
| Late (≥30 wks) pregnancy | 67 (94.4) | 5 (41.7) | 261 (40.8) | 27 (7.1) |
| Hb>13 g/dl, % |  |  |  |  |
| Early (≤20 wks) pregnancy | 0 (0.0) | 0 (0.0) | 36 (5.5) | 152 (39.8) |
| Mid (21-29 wks) pregnancy | 0 (0.0) | 1 (9.1) | 4 (0.6) | 53 (14.0) |
| Late (≥30 wks) pregnancy | 0 (0.0) | 1 (8.3) | 17 (2.7) | 93 (24.5) |

^1^ Values are means (SDs) or n (%), BMI: body mass index; FA: Folic Acid; Hb: Hemoglobin; IFA: Iron and Folic Acid; MM: Multiple Micronutrient; SGA: small for gestational age.

**OSM Table 2: Baseline characteristics of included and excluded participants at preconception enrollment**

| **Variable** | **Pregnancy Analysis**  **(n=1175)** | **Excluded sample**  **(n=404)** | **P-value** |
| --- | --- | --- | --- |
| Age, *y* | 26.0 ± 4.3 | 25.6 ± 4.4 | 0.12 |
| Minority ethnic, *%* | 577 (49.2) | 201 (49.8) | 0.85 |
| Education Level, *%* |  |  |  |
| Primary school | 88 (7.5) | 41 (10.1) | 0.35 |
| Secondary school | 636 (54.2) | 218 (54.0) |  |
| High school | 305 (26.0) | 95 (23.5) |  |
| College or higher | 145 (12.4) | 50 (12.4) |  |
| Work as farmers, *%* | 938 (79.9) | 320 (79.2) | 0.77 |
| Socio-economic status, *%* |  |  |  |
| Low | 385 (32.8) | 138 (34.3) | 0.84 |
| Average | 393 (33.5) | 133 (33.1) |  |
| High | 396 (33.7) | 131 (32.6) |  |
| Number of children ≥ 1, *%* | 927 (94.2) | 334 (95.7) | 0.29 |
| Preconception Nutritional status |  |  |  |
| Weight, *kg* | 45.7 ± 5.3 | 46.0 ± 5.8 | 0.47 |
| Height, c*m* | 152.6 ± 5.0 | 153.0 ± 5.2 | 0.20 |
| BMI, *kg/m^2^* | 19.6 ± 2.0 | 19.6 ± 2.1 | 0.95 |
| Low BMI (<18.5), *%* | 359 (30.6) | 126 (31.5) | 0.75 |
| Preconception Hb, *g/dl* | 12.9 ± 1.3 | 12.9 ± 1.3 | 0.88 |
| Anemia, % (Hb <12 g/dL) | 236 (20.1) | 77 (19.4) | 0.76 |

^1^ Values are means (SDs) or n (%), BMI: body mass index; FA: Folic Acid; Hb: Hemoglobin; IFA: Iron and Folic Acid; MM: Multiple Micronutrient; SGA: small for gestational age.

**OSM Table 3: Child characteristics of included and excluded participants**

| **Variable** | **Pregnancy Analysis (n=1175)** | **Excluded sample**  **(n=404)** | **P-value** |
| --- | --- | --- | --- |
| **Child Characteristics at Birth** | | | |
| Female, *%* | 584 (49.7) | 197 (48.8) | 0.73 |
| Gestational age, *wk* | 39.3 ± 1.9 | 38.7 ± 2.4 | <0.001 |
| Preterm, *%* | 100 (8.5) | 56 (14.6) | <0.001 |
| Birth weight, *gr* | 3094.4 ± 439.7 | 3049.6 ± 445.1 | 0.081 |
| Low birth weight, *%* | 52 (4.4) | 21 (5.3) | 0.47 |
| SGA, *%* | 172 (15.5) | 42 (12.7) | 0.21 |
| Birth length, cm | 48.9 ± 3.1 | 49.0 ± 2.5 | 0.66 |
| **Child Hb** | | | |
| Hb at 3 mo, *g/dl* | 10.6 ± 1.3 | 10.5 ± 1.3 | 0.22 |
| Anemia, % (Hb <11 g/dL) | 614 (61.0) | 206 (64.2) | 0.30 |
| Hb at 6 mo, *g/dl* | 10.5 ± 1.1 | 10.6 ± 1.2 | 0.28 |
| Anemia, % (Hb <11 g/dL) | 636 (66.3) | 176 (63.3) | 0.36 |
| Hb at 12 mo, *g/dl* | 10.8 ± 1.2 | 10.7 ± 1.2 | 0.35 |
| Anemia, % (Hb <11 g/dL) | 499 (52.9) | 149 (54.0) | 0.75 |
| Hb at 24 mo, g/dl | 11.2 ± 1.1 | 11.1 ± 1.2 | 0.50 |
| Anemia, % (Hb <11 g/dL) | 424 (41.1) | 134 (42.9) | 0.57 |
| **Bayley scales for infant development at 12 mo** | | | |
| Cognitive | 112.2 ± 10.3 | 112.0 ± 10.3 | 0.74 |
| Language | 97.8 ± 11.0 | 96.8 ± 11.3 | 0.14 |
| Motor | 102.9 ± 11.7 | 102.5 ± 11.0 | 0.52 |
| Bayley scales (overall) | 104.3 ± 8.7 | 103.7 ± 8.4 | 0.31 |
| **Bayley scales for infant development at 24 mo** | | | |
| Cognitive | 99.3 ± 9.8 | 100.4 ± 10.4 | 0.081 |
| Language | 102.2 ± 11.0 | 102.7 ± 10.5 | 0.49 |
| Motor | 105.8 ± 12.1 | 105.5 ± 11.0 | 0.70 |
| Bayley scales (overall) | 102.9 ± 8.9 | 102.4 ± 9.2 | 0.44 |
| **Wechsler scale global intelligence, performance and verbal scores at 6-7 y** | | | |
| Verbal comprehension index (VCI) | 82.0 ± 12.4 | 81.1 ± 12.4 | 0.27 |
| Perceptual reasoning index (PRI) | 93.5 ± 14.2 | 91.8 ± 15.1 | 0.068 |
| Working memory index (WMI) | 101.8 ± 11.6 | 101.9 ± 11.2 | 0.83 |
| Processing speed index (PSI) | 89.6 ± 12.4 | 88.6 ± 11.9 | 0.24 |
| Full scale IQ (FSIQ) | 88.6 ± 12.2 | 87.5 ± 11.9 | 0.17 |

^1^ Values are means (SDs) or n (%), Hemoglobin: Hb
